# When and How Do Soccer Players From a Semi-Professional Club Sprint in Match Play?

**DOI:** 10.5114/jhk/159964

**Published:** 2023-01-20

**Authors:** José M. Oliva-Lozano, Juan Cuenca-López, Javier Suárez, Paulino Granero-Gil, José M. Muyor

**Affiliations:** 1 Health Research Centre, University of Almería, Almería, Spain.; 2 Club Polideportivo El Ejido, Almería, Spain.; 3 Ferencvárosi Torna Club, Budapest, Hungary.; 4 Laboratory of Kinesiology, Biomechanics and Ergonomics (KIBIOMER Lab), Research Central Services, University of Almería, Almería, Spain.

**Keywords:** football, high-speed running, performance, GPS, team sport, high-intensity efforts

## Abstract

The aims of this study were to investigate the periods in which sprints occurred during official matches and analyze these sprints considering the effect of the playing position and different contextual variables. Electronic performance and tracking systems were used for the analysis of all sprints performed by players. Matches were recorded by video and synchronized with performance tracking data. A total of 252 sprints were analyzed. The greatest frequency of sprints was observed in the period 1 (0’–15’), followed by period 2 (15’–30’) and period 6 (75’–90’), regardless of the playing position (χ^2^ = 31.35; p = 0.051). Most sprints were non-linear (non-linear sprints: 97.6%; linear sprints: 2.4%) and without ball possession (without ball possession: 95.2%; with ball possession: 4.8%) for all playing positions, but the role of the sprint and the field area in which the sprint occurred were dependent on the position (p < 0.001). Specifically, players covered ~17.55 m per sprint, starting at ~10.34 km/h, reaching ~26.74 km/h, maximally accelerating at ~2.73 m/s^2^, and decelerating at ~3.61 m/s^2^. Overall, the playing position and contextual variables had no significant effect on physical performance variables analyzed during these sprints. Therefore, this study allows performance practitioners to have a better understanding of when and how soccer players sprint in match-play. In this regard, this study presents some training and testing strategies that may be considered to improve performance and decrease injury risk.

## Introduction

During the past decade, the use of electronic performance and tracking systems in the context of team sports has allowed a better understanding of players’ physical performance (Pino-Ortega et al., 2021). Based on positioning and time, variables related to velocity, acceleration, and distance covered may be collected and thus, most tracking systems are based on global positioning systems (GPS), optical tracking systems, and local positioning systems (Oliva-Lozano and Muyor, 2021; Pino-Ortega et al., 2021). These data may be used across a myriad of applications (e.g., monitoring and planning external loads), which allows an objective decision-making process regarding performance and injury risk (TorresRonda et al., 2022).

Soccer is a team sport with a locomotive intermittent profile, which is characterized by repeated high-intensity actions that are interspersed with recovery periods of lower intensity (Oliva-Lozano, Barbier, et al., 2021). However, an increase in high-speed running demands has been observed in soccer matches in recent years (Pons et al., 2021). In addition, previous research found that sprints could be considered a prerequisite for successful performance provided that linear sprints are the most frequent actions in goal situations (Faude et al., 2012), evading an opponent or creating a shot (Sweeting et al., 2017). In consequence, practitioners should design training drills that improve sprinting capabilities (Beato et al., 2021; Haugen et al., 2014; Oliva-Lozano, Fortes, et al., 2020).

In this regard, previous research has focused on physical performance analysis of sprinting actions. For example, a recent study concluded that soccer players perform ~10 sprints per match and cover ~19.5 m per sprint (OlivaLozano, Fortes, et al., 2020), although there may be differences according to the playing position (Ingebrigtsen et al., 2015; Oliva-Lozano, Fortes, et al., 2020). From a practical perspective, these data are useful for understanding the demands of match play, but have limited application for training purposes because it is unknown how players perform those actions.

However, an integrated approach that contextualizes sprint performance may allow progress towards the understanding of global demands (Bradley and Ade, 2018; Ju et al., 2022; Oliva-Lozano, Fortes, et al., 2021; Oliva-Lozano, Martínez-Puertas, et al., 2021). It is important to know when and how players experience these sprints (e.g., period of the match, sprint with or without the ball, sprint trajectory, or role of the sprint) (Oliva-Lozano, Fortes, et al., 2021) since this has key implications for performance practitioners. For instance, the player’s fatigue is not similar when experiencing sprints above 24 km/h in the first minutes of the match compared to sprints in the last minutes of the match. To date, research on the context of sprint performance is scarce at any soccer level, but to the best of the authors’ knowledge, it is unknown when and how players from a semi-professional soccer club sprint in match-play. Consequently, the aims of this study were to: 1) investigate the periods in which sprints occurred during official matches; and 2) analyze these sprints considering the effect of the playing position and different contextual variables.

## Methods

### Study Design

Electronic performance and tracking systems, based on a global positioning system (GPS), were used for the analysis of all sprints (above 24 km/h) performed by players during 6 matches. These matches were recorded by video and then, synchronized with the performance tracking data.

### Participants

A total of 20 male soccer players (age: 23.0 ± 5.3 years old; body height: 1.79 ± 0.06 m; body mass: 73.9 ± 3.7 kg) from a Spanish semi-professional club participated in the study. Players were categorized based on the playing position (FW: forwards; WMF: wide midfielders; MF: midfielders; CD: central defenders, FB: full backs) and a total of 252 sprints were registered. Goalkeepers were not included in the study because of the different nature of their activity-profile (Oliva-Lozano, Fortes, et al., 2021; White et al., 2018). The inclusion criterion was that the player had to complete the total duration of the match. Participants provided informed consent for the use of their data for the study. Also, the club approved the data collection during official competition and the study was authorized by the Bioethics Committee of the University of Almeria (UALBIO2020/032).

### Procedures

Players worn WIMU Pro systems (RealTrack Systems, Almeria, Spain), which are based on inertial sensors and GPS technology and are valid and reliable instruments for the analysis of physical performance variables (Bastida-Castillo et al., 2018). These instruments have been approved by the FIFA Quality Program as well (FIFA, 2020; Oliva-Lozano and Muyor, 2021). All tracking systems were calibrated according to the manufacturer’s instructions 30 min before the start of the match. Then, tracking systems were placed in a chest vest (Rasán, Valencia, Spain), which was given to each player.

In addition, SPro software (RealTrack Systems, Almeria, Spain) was used to synchronize the video with physical performance data. According to a previous study with similar aims (Oliva-Lozano, Fortes, et al., 2021), the following variables from the Intervals Pro report in SPro (RealTrack Systems, Almeria, Spain) were downloaded for each sprint above 24 km/h: maximum velocity (Vmax, km/h), starting velocity (Vo, km/h), distance covered sprinting (SPD, m), maximum acceleration (ACCmax, m/s^2^), and maximum deceleration (DECmax, m/s^2^). Since the video was synchronized with the data, the following contextual variables were included based on an observational analysis (Oliva-Lozano, Fortes, et al., 2021): ball possession (i.e., sprints with/without the ball), trajectory (i.e., linear/nonlinear sprints), role of the sprint (i.e., offensive/defensive sprints) and field area in which the sprint occurred (i.e., sprint in own/the opponent team’s field area). Moreover, each sprint was categorized within one of the 15 min periods: period 1 (1’–15’), period 2 (15’–30’), period 3 (30’– 45’), period 4 (45’–60’), period 5 (60’–75’), and period 6 (75’–90’) given that the first aim of this study was to examine the periods in which sprints occurred in the course of the game.

### Statistical Analysis

First of all, descriptive statistics were calculated to find the periods in which sprints occurred in match-play. Then, the Chi-Squared test was performed to analyze the association between the playing position and the periods in which the sprint occurred. The Pearson Chi-Squared statistic (χ^2^) was selected, but if 20% of the categories (at least) had expected frequencies lower than 5, the likelihood ratio (LR) statistic was considered (Koehler and Larntz, 1980). If the association between the playing position and the periods in which sprints occurred was not significant, the null hypothesis for the independence of variables was accepted at the 95% confidence level. However, if the association between the variables was significant, the null hypothesis was rejected and adjusted standardized residuals were calculated to investigate among which categories there were large differences (Oliva-Lozano, Fortes, et al., 2021). An adjusted residual with an absolute value higher than 1.96 showed that the number of cases within that cell was significantly greater or lower than would be expected if the null hypothesis were true. Furthermore, Crammer’s V was considered as a measure of effect size (ES) for the chi-squared test.

Considering the second aim of the study, the Chi-Squared test was used with the same criteria as the first aim to analyze the association between the playing position and contextual variables of sprints. Nevertheless, a multi-factor analysis of variance through a general linear model was also carried out to analyze the effect that the contextual variables (i.e., ball possession, trajectory, role, and field area in which the sprint occurred) and playing position (i.e., FW, WMF, MF, CD, and FB) had on sprint-related physical performance (i.e., Vo, Vmax, SPD, ACCmax, and DECmax). In this regard, contextual variables and the playing position were set as independent variables while physical performance variables were dependent variables. Then, Bonferroni post hoc tests were carried out to compare between independent variables. In this case, the effect sizes were reported using partial eta-squared (ηp^2^). All the analyses were conducted using SPSS Statistics for Windows version 27 (IBM Corp., Armonk, NY, USA) with a level of significance at *p* ≤ 0.05. Nevertheless, statistical power, which was greater than 0.85 in all the variables analyzed for the sample size of the study, was calculated using G*Power software (Heinrich-Heine-Universität Düsseldorf, Düsseldorf, Germany) (Faul et al., 2007).

## Results

**Figure 1:**
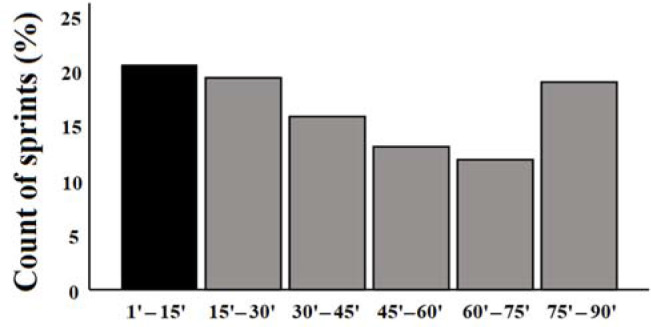
. Total of sprints (%) registered for each 15-min period of match play.

Figure 1 shows the total of sprints registered for each 15-min period of match play. The greatest frequency of sprints was observed in the period 1 (0’–15’: 20.6% of cases), followed by period 2 (15’–30’: 19.4% of cases) and period 6 (75’– 90’: 19% of cases). In addition, the playing position had no significant effect on the period in which sprints occurred (χ^2^ = 31.35; *p* = 0.051; ES = 0.18), thus this implies the acceptance of the null hypothesis for the independence of variables at the 95% confidence level.

Table 1 shows the total of sprints by the playing position and contextual variable. The results indicate that there was no significant effect of the playing position on ball possession (sprints with ball possession: 4.8% of cases; sprints without ball possession: 95.2% of cases; LR = 7.39; *p* = 0.12; ES = 0.20) or sprint trajectory (non-linear sprints: 97.6% of cases; linear sprints: 2.4% of cases; LR = 5.74; *p* = 0.22; ES = 0.13). However, there was a significant effect of the playing position on the role of the sprint (χ^2^ = 64.79; *p* < 0.001; ES = 0.51) and field area in which the sprint occurred (χ^2^ = 75.48; *p* < 0.001; ES = 0.55), which explains the rejection of the null hypothesis for the independence of the variables at the 95% confidence level. Specifically, there was a significant association between the role of the sprint and the following playing positions: CD (offensive sprints = 7.06%; defensive sprints = 92.94%; adjusted standardized residual = 5.3), WMF (offensive sprints = 52.17%; defensive sprints = 47.83%; adjusted standardized residual = 2.7), and FB (offensive sprints = 25.71%; defensive sprints = 74.29%; adjusted standardized residual = 6.6). Moreover, there was a significant association between the field area in which the sprint occurred and CD (sprints in own field: 87.06%; sprints in the opponent field: 12.94%; adjusted standardized residual = 6.4), MF (sprints in own field: 43.14%; sprints in the opponent field: 56.86%; adjusted standardized residual = 2.6), WMF (sprints in own field: 17.39%; sprints in the opponent field: 82.61%; adjusted standardized residual = 4.3), and FW (sprints in own field: 8.69%; sprints in the opponent field: 8.69%; adjusted standardized residual = 5.2).

**Table 1:** Descriptive statistics for sprints registered in match play considering each contextual variable and playing position.

Variables			CD	FB	MF	WMF	FW	ALL	χ2	*p*	ES
Ball possession	Yes	4.71%		1.43%	3.92%	4.35%	17.39%	4.76%	7.39*	0.117	0.20
	No	95.29%		98.57%	96.08%	95.65%	82.61%	95.24%			
Trajectory	Linear	4.71%		1.43%	0.00%	4.35%	0.00%	2.38%	5.74	0.220	0.13
	Non-linear	95.29%		98.57%	100.00%	95.65%	100.00%	97.62%			
Role	Offensive	7.06%		25.71%	29.41%	52.17%	86.96%	28.17%	64.79	0.001	0.51
	Defensive	92.94%		74.29%	70.59%	47.83%	13.04%	71.83%			
Field area	Own team	87.06%		67.14%	43.14%	17.39%	8.70%	59.13%			
	Opponent team	12.94%		32.86%	56.86%	82.61%	91.30%	40.87%	75.48	0.001	0.54

Note: χ^2^ = chi squared; *Chi squared statistic presented as the likelihood ratio (LR) given that at least 20% of the categories had expected frequencies lower than 5; ES = effect size; CD = central defender; FB = full back; MF = midfielder; WMF = wide midfielder; FW = forward

**Figure 2:**
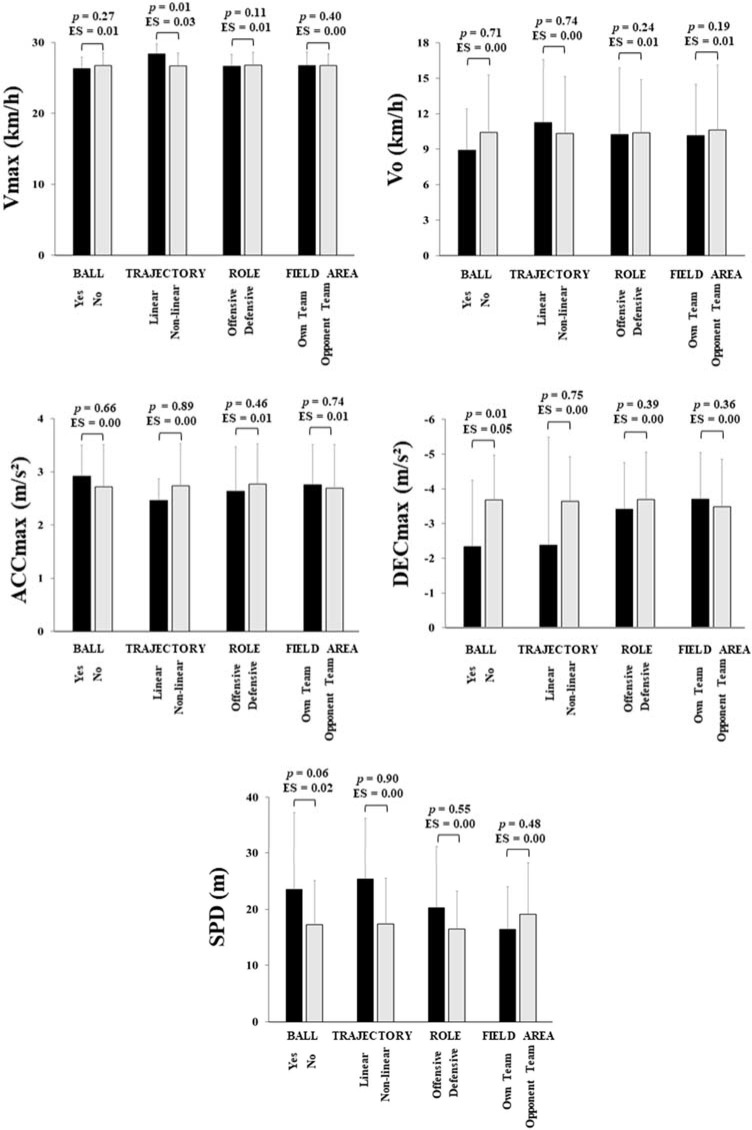
. Physical performance required by sprints for each contextual variable. Note: V_max_ = maximum velocity; V_o_ = starting velocity; ACC_max_ = maximum acceleration; DEC_max_ = maximum deceleration; SPD: sprinting distance.

Figure 2 shows physical performance variables analyzed during these sprinting actions. The results showed that semi-professional soccer players covered ~17.55 m per sprint, starting at ~10.34 km/h, reaching ~26.74 km/h, maximally accelerating at ~2.73 m/s^2^, and maximally decelerating at ~3.61 m/s^2^. The playing position had no significant effect on physical performance variables analyzed during these sprinting actions (F = 0.48–0.76; *p* > 0.05; ES = 0.01). In addition, none of the contextual variables (i.e., sprint trajectory, ball possession, role of the sprint, and field area in which the sprint occurred) had a significant effect on physical performance variables (F = 0.02–3.67; *p* > 0.05; ES = 0.00–0.01), except ball possession on DECmax (sprints without the ball: ~3.68 m/s^2^ > sprints with the ball: ~2.33 m/s^2^; F = 10.77; *p* ≤ 0.01; ES = 0.05) and sprint trajectory on Vmax (linear sprints: ~28.37 km/h > non-linear sprints: ~26.70 km/h; F = 6.02; *p* ≤ 0.01; ES = 0.03). The interaction between the playing position and each contextual variable was not significant for all of the physical performance variables (F = 0.07–2.45; *p* > 0.05; ES = 0.01–0.03).

## Discussion

The main aim of this study was to investigate when and how soccer players from a semi-professional club sprinted in match play. The main findings were that: a) the greatest frequency of sprints was observed in the 1^st^ period (0’–15’), followed by the 2^nd^ period (15’–30’) and the 6^th^ period (75’–90’), regardless of the playing position; b) most sprints were non-linear and without ball possession for all playing positions, but the role of the sprint and the field area in which the sprint occurred were dependent on the position; c) players covered ~17.55 m per sprint, starting at ~10.34 km/h, reaching ~26.74 km/h, maximally accelerating at ~2.73 m/s^2^, and maximally decelerating at ~3.61 m/s^2^; d) overall, the playing position and contextual variables had no significant effect on physical performance variables analyzed during these sprinting actions.

This study observed that the first period of the match elicited the greatest amount of sprints. This is consistent with previous research (OlivaLozano, Fortes, et al., 2021), which found that maximum speed actions were performed in the first 15 min of each match half by professional soccer players, regardless of the playing position. Nonetheless, to the best of the authors’ knowledge this is the first study in a semi-professional soccer club. In addition, a previous study analyzing when the most demanding passages of play occurred in professional soccer players found that the first 15 min of the match showed the greatest amount of passages (Oliva-Lozano, Martínez-Puertas, et al., 2021). Moreover, our results suggest that sprint performance is reduced towards the end of the match, which may be due to the influence of fatigue as glycogen levels are reduced (Krustrup et al., 2006; Oliva-Lozano, Fortes, et al., 2021; Reilly et al., 2008). However, the last period of the match reported an increase in the total amount of sprints compared to the 3^rd^ (30’–45’), 4^th^ (45’–60’), and 5^th^ (60’–75’) period, which may be related to the effect of other situational variables such as match status or score-line (Trewin et al., 2017).

Another finding of this study, which is line with a previous study (Oliva-Lozano, Fortes, et al., 2021), was that most sprints were non-linear and without ball possession. For example, OlivaLozano et al. (2021) found that 94.5% of sprints were without ball possession and 70.3% were nonlinear sprints while our study showed that 95.2% of sprints were without ball possession and 97.6% of sprints were non-linear. These findings have important implications for strength and conditioning coaches when designing not only training drills but also testing sessions (e.g., curve sprint test) (Fílter et al., 2020; Oliva-Lozano, Fortes, et al., 2021). In addition, the results showed that the role of the sprint and the field area in which the sprint occurred was dependent on the playing position as found in previous studies (Andrzejewski et al., 2015; Oliva-Lozano, Fortes, et al., 2021). For instance, a previous study observed that positions such as WMF and FW showed greater offensive than defensive sprints (FW = ~20.6 vs. ~4.9 sprints; WMF = ~15.9 vs. ~8.7 sprints) (Andrzejewski et al., 2015). This suggests that the role of the sprint and the field area in which the sprint occurred is related to the technical/tactical role of these playing positions (e.g., FW usually reach the opponent’s penalty area or sprint for taking a shot on goal, but they are not usually involved in team’s defensive actions) (Andrzejewski et al., 2014; Oliva-Lozano, Fortes, et al., 2021).

When it comes to physical performance required by these sprinting actions, it was observed that the playing position and contextual variables had no significant effect on physical performance variables in general. Only a significantly greater DEC_max_ was found in sprints without the ball compared to sprints with the ball and greater V_max_ in linear sprints compared to nonlinear sprints, which are reasonable results considering the nature of these actions. However, the fact that the playing position had no significant effect on sprint-related performance variables is not in line with previous studies (Andrzejewski et al., 2015; De Hoyo et al., 2018; Oliva-Lozano, Fortes, et al., 2020, 2020, 2021). For example, we found that players covered ~17.6 m per sprint while top level Europa League players covered ~21 m per sprint, being the greatest SPD covered by FB (~22 m) and the lowest SPD covered by MF (~19.7 m) (Andrzejewski et al., 2015). Also, a recent study found that the SPD was similar to our study (~19.5 m) and positional differences were observed between CD (~16.9 m) and WMF (~21.6 m) (OlivaLozano, Fortes, et al., 2020). Another study analyzing V_max_ from maximum speed actions registered in match play found that MF got significantly lower V_max_ (~28.34 km/h) compared to the rest of playing positions (WMF = ~31.94 km/h; FB = ~30.57 km/h; CD = ~30.41 km/h; FW = ~30.13 km/h) (Oliva-Lozano, Fortes, et al., 2021). Although it may be observed that those values of V_max_ are greater compared to our study, this may be due to the fact that we averaged V_max_ from all actions above 24 km/h while Oliva-Lozano et al. (2021) averaged data from maximum speed actions only. However, that study concluded that there was no significant effect from any contextual variable on Vo, ACCmax, or DECmax. In this regard, our study is consistent with previous research considering that players usually sprint from low intensity running speeds (e.g., above 7–10 km/h), maximally accelerating at ~3 m/s^2^ and decelerating at ~4 m/s^2^ (Oliva-Lozano, Fortes, et al., 2021).

Finally, it is important to mention that this study has some limitations. For instance, only data from 20 players were analyzed and future studies might include larger sample size even though this is not easy in the context of semi-professional and professional soccer. Also, other speed thresholds for sprint calculation could have been analyzed (e.g., 25.2 km/h or 30 km/h) since this may have an impact on the sprint demands (e.g., greater speeds thresholds may be more difficult to reach and may require more distance to cover compared to lower speed thresholds). Moreover, situational variables (e.g., match status or match location) were not considered while these might have an impact on players’ performance (Oliva-Lozano, RojasValverde, et al., 2020; Trewin et al., 2017). In addition, this study included GPS technology while future studies could include new tracking systems to improve accuracy of the data collected (e.g., tracking systems ultra-wide band technology) (Pino-Ortega et al., 2021).

In conclusion, this study allows strength and conditioning coaches to have a better understanding of when and how soccer players sprint in match play. Since the greatest frequency of sprints was observed in the 1^st^ period (0’–15’), followed by the 2^nd^ (15’–30’) and the 6^th^ period (75’–90’), training drills need to be designed in order to maximize performance in these periods of the match. Specifically, warm-up strategies need to prepare players to perform high-speed running actions in the first stages of the match. Nonetheless, resistance to fatigue needs to be trained since frequency of sprints tends to decrease towards the end of the match and the last 15-min period of the match showed an increase in the number of sprints.

Also, training drills and testing sessions need to consider that most sprints were non-linear and without ball possession. For instance, this implies that using novel methods for sprint training (e.g., repeated-sprint training including changes of direction) (Beato et al., 2019; López-Segovia et al., 2014) or sprint testing such as the curve sprint test is highly recommended (Fílter et al., 2020). Considering that the role of the sprint and the field area in which the sprint occurred was dependent on the playing position, training sessions may be adapted according to these findings. For example, a 3 vs. 3 drill may be designed in which defensive players need to perform a recovery run (~20–30 m) to secure the team’s goal area while offensive players try to reach the opponent’s area and sprint for taking a shot on goal. Moreover, a recent study suggested the use of medium-sided games and large-sided games with ~300 m^2^ of player density in order to efficiently develop and maintain high-speed running and sprinting capabilities (Beato et al., 2021). Also, sprinting and testing drills might be performed with Vo around 10 km/h. Therefore, this may allow players to cover similar SPD (~18 m per sprint), Vo (~10 km/h), V_max_ (~27 km/h), ACC_max_ (~2.73 m/s^2^) and DEC_max_ (~3.61 m/s^2^) to match demands. All these training strategies may be considered to improve performance and decrease injury risk. Regardless of the population characteristics, sprint performance can be improved by increasing the orientation and/or magnitude of force that a player can generate and express in the sprint (Nicholson et al., 2021, 2022).
